# *In Situ* Monitoring of Temperature inside Lithium-Ion Batteries by Flexible Micro Temperature Sensors

**DOI:** 10.3390/s111009942

**Published:** 2011-10-21

**Authors:** Chi-Yuan Lee, Shuo-Jen Lee, Ming-Shao Tang, Pei-Chi Chen

**Affiliations:** Department of Mechanical Engineering, Yuan Ze Fuel Cell Center, Yuan Ze University, 135 Yuan-Tung Road, Chung-Li, Taoyuan 32003, Taiwan; E-Mails: mesjl@saturn.yzu.edu.tw (S.-J.L.); gefb3954@gmail.com (M.-S.T.); peggy416420@gmail.com (P.-C.C.)

**Keywords:** MEMS, micro temperature sensor, lithium-ion secondary battery, *in situ* monitoring

## Abstract

Lithium-ion secondary batteries are commonly used in electric vehicles, smart phones, personal digital assistants (PDA), notebooks and electric cars. These lithium-ion secondary batteries must charge and discharge rapidly, causing the interior temperature to rise quickly, raising a safety issue. Over-charging results in an unstable voltage and current, causing potential safety problems, such as thermal runaways and explosions. Thus, a micro flexible temperature sensor for the in *in-situ* monitoring of temperature inside a lithium-ion secondary battery must be developed. In this work, flexible micro temperature sensors were integrated into a lithium-ion secondary battery using the micro-electro-mechanical systems (MEMS) process for monitoring temperature *in situ*.

## 1. Introduction

In recent years, lithium-ion secondary batteries have been extensively used in commercial products, such as smart phones, personal digital assistants (PDA), notebooks and electric cars. Given this widespread use, the safety and efficiency of lithium-ion secondary batteries are important issues.

The safety of a lithium-ion secondary battery depends on the electrolyte, separator, anode and cathode [1]. Under overcharge conditions, lithium forms an active surface and reacts with the electrolyte, increasing internal impedance and reducing discharge efficiency. With increasing charge-discharge cycles, the capacity of the battery will decrease, limiting its cycle life.

Metallic lithium can separate out in the form of dendrite and acicular crystals and cause many problems during rapid charging and discharging [2]. Accordingly, the efficiency of the battery is reduced, and a problem of safety arises [3,4]. The internal temperature of a lithium-ion secondary battery is typically measured using a thermocouple. Traditional thermocouples are too large to be able to be used to accurately determine the temperature at an optimal measurement position [5]. Damage to the battery during temperature measurement should be avoided. A four-probe electrical technique that is both fast (<200 ms) and accurate (±0.1 °C) and works by connecting to the two terminals of the cell [6], eliminates the need for inserting any probe into the cell. Remote query sensors are used to measure the temperature, pressure, fluid-flow velocity and humidity [7–9]. In this investigation, resistance temperature detector (RTD) micro temperature sensors with small volume, high accuracy, short response time, simplicity of fabrication, mass-producibility, and the capacity to measure the temperature more effectively than traditional thermocouples, are utilized. These micro temperature sensors must be resistant to erosion, high temperature and stress corrosion.

## 2. Methodology

### 2.1. Design of a Micro Temperature Sensor

**Figure 1.**
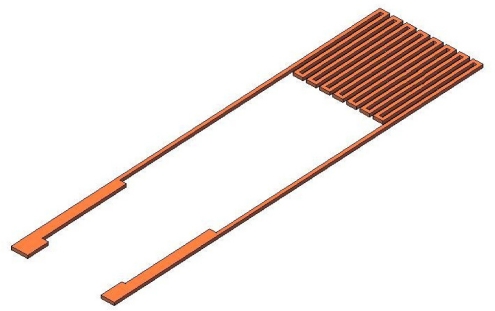
Schematic diagram of a micro temperature sensor.

Temperature sensors fall into four main classes—gold RTDs, thermally sensitive resistors (thermistors), thermocouples, and mercury-in-glass thermometers. The micro temperature sensor that is fabricated herein is a gold RTD, and is illustrated in Figure 1.

The resistance of a general metal is given by:

1 R=ρL/A

where *R* denotes resistance (Ω); *ρ* presents resistivity (Ωm); *L* denotes wire length (m), and *A* denotes cross-sectional area (m^2^). When the temperature of the RTD varies in the linear region, the relationship between the measured resistance and the change in temperature can be expressed as:

2 $$R_{t}=R_{i}(1+\alpha_{t}\Delta T)$$

3 $$\Delta T=t-t_{i}$$

where *R_t_* and *R_i_* represent the resistance of an RTD at *t* °C and *t_i_* °C, respectively; *α_t_* represents the positive temperature coefficient of an RTD; Δ*T* denotes the variation in temperature from the reference temperature; and *t* and *t_i_* are the temperature of an RTD at *t* °C and *t_i_* °C, respectively. Equation (2) can thus be rewritten as

4 $$\alpha_{t}=\frac{R_{t}-R_{i}}{R_{i}(\Delta T)}$$

where *α_t_* is the temperature coefficient of resistance (TCR) of the sensor.

### 2.2. Fabrication of Micro Temperature Sensor

**Figure 2.**
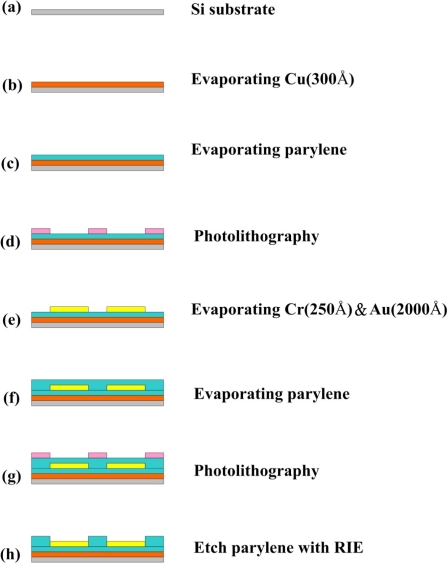
Procedure for fabricating flexible micro temperature sensors on silicon substrate.

In this study, parylene, in the form of a thin film, was adopted as a flexible micro temperature sensor. Parylene is resistant to erosion and stress corrosion. The sensor material was deposited layer-by-layer by the physical vapor deposition (PVD) technique; thus, the micro temperature sensors could be fabricated at low temperature with accurately controlled thickness. Figure 2 presents the eight steps (a–h) involved in fabricating the sensor.

**Figure 3.**
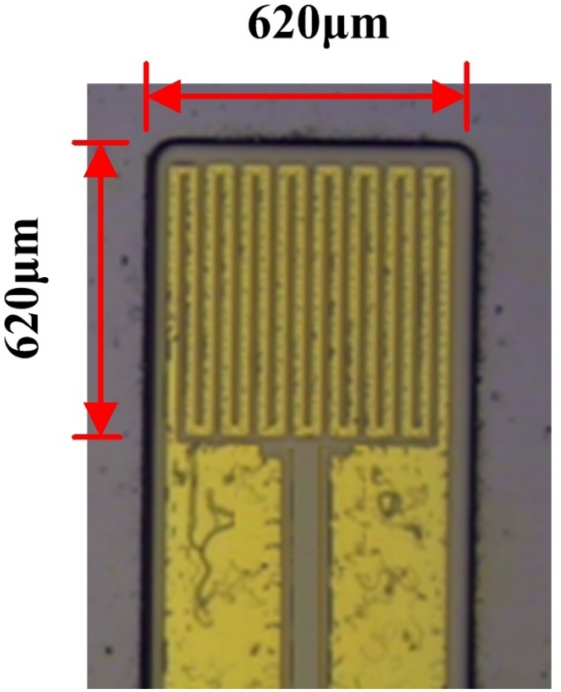
Optical microscopic photograph of the flexible micro temperature sensor.

Steps a and b: a layer of copper (Cu) was deposited as a sacrificial layer on a silicon wafer substrate, and then a 300 Å-thick parylene thin film was deposited onto the layer of copper, as displayed. Step c: the parylene thin film acted as a protective layer, an isolation layer and a substrate. Step d: the first lithographic process was carried out with the purpose of defining the pattern on the micro temperature sensors. Step e: Cr (250 Å) and then Au (2000 Å) were deposited on the parylene substrate as a conduction layer using an e-beam evaporator. The structure of the micro temperature sensors was formed with the lift-off process. Step f: then, another parylene layer was deposited to protect the micro temperature sensors. Steps g and h: the second lithographic process, reactive ion etching (RIE), defined the pattern on the contact pads and in the sensing region. Figure 3 shows an optical microscope (OM) image of the micro temperature sensor.

## 3. Results and Discussion

### 3.1. Calibration of Micro Temperature Sensors

**Figure 4.**
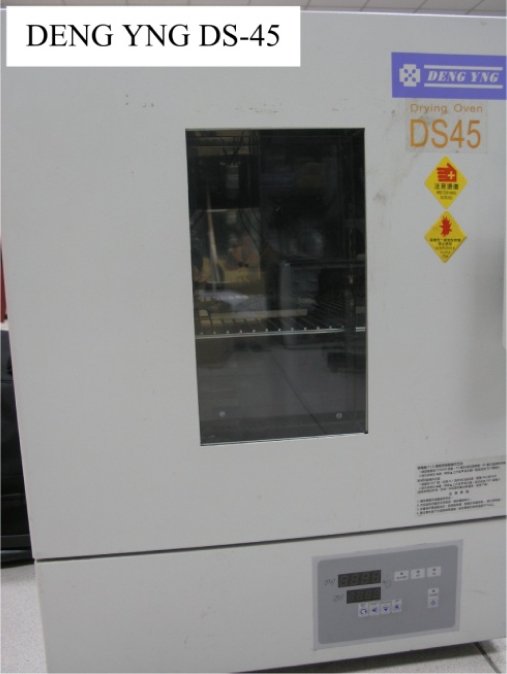
Calibration of micro temperature sensors in a thermal chamber.

The micro temperature sensor was placed in a thermal chamber (DENG YNG DS-45), as presented in Figure 4.

**Figure 5.**
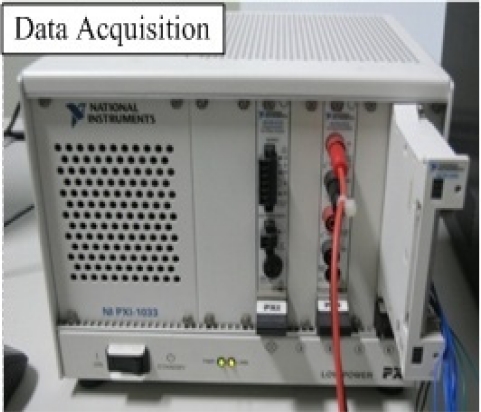
Resistance signal is picked up by Data Acquisition.

The resistance signal was picked up by a Data Acquisition system, as displayed in Figure 5. The temperature of the thermal chamber was increased from −20 °C to 90 °C three times.

**Figure 6.**
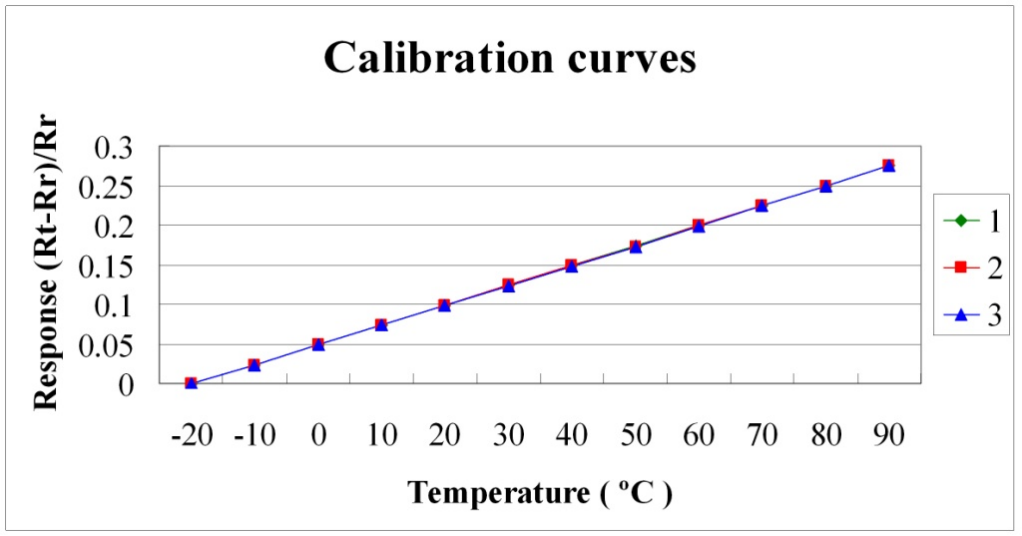
Calibration curves of sensor 1.

**Figure 7.**
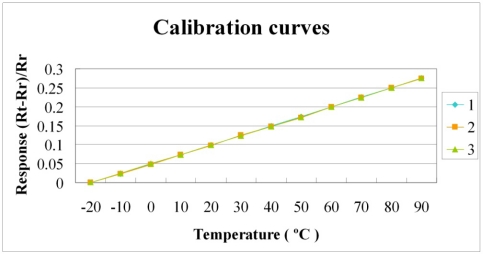
Calibration curves of sensor 2.

Figures 6 and 7 plot the calibration curves of the micro temperature sensor. The calibration curve exhibits high repeatability and linearity of the relationship between temperature and resistance.

### 3.2. Thermal Shock Test

**Figure 8.**
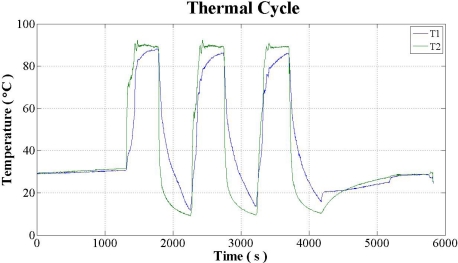
Temperature change of space in Programmable Thermal Shock Tester and holder of micro temperature sensor 1.

**Figure 9.**
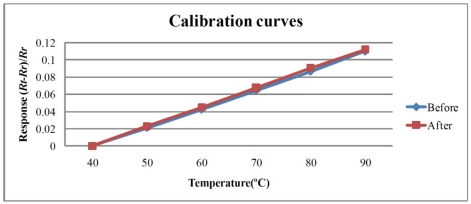
Comparison of micro temperature sensor 1 calibration curves before and after thermal shock testing for three cycles.

An experiment was conducted to test the strength of the micro temperature sensor structure using a Programmable Thermal Shock Tester wherein a sensor placed inside the tester measured and reported the temperature. The temperatures were cycled three times between 0 °C and 90 °C by ramping the temperatures over a period of 3 minutes with a 5 minutes dwell-time at each extreme temperature. In Figure 8, T1 is the temperature curve of space in the Programmable Thermal Shock Tester, T2 is the temperature curve of holder placed micro temperature sensor 1. Figure 9 plots the calibration curve of micro temperature sensor 1.

### 3.2. Temperature Measurement in 1C Charging and Discharging

**Figure 10.**
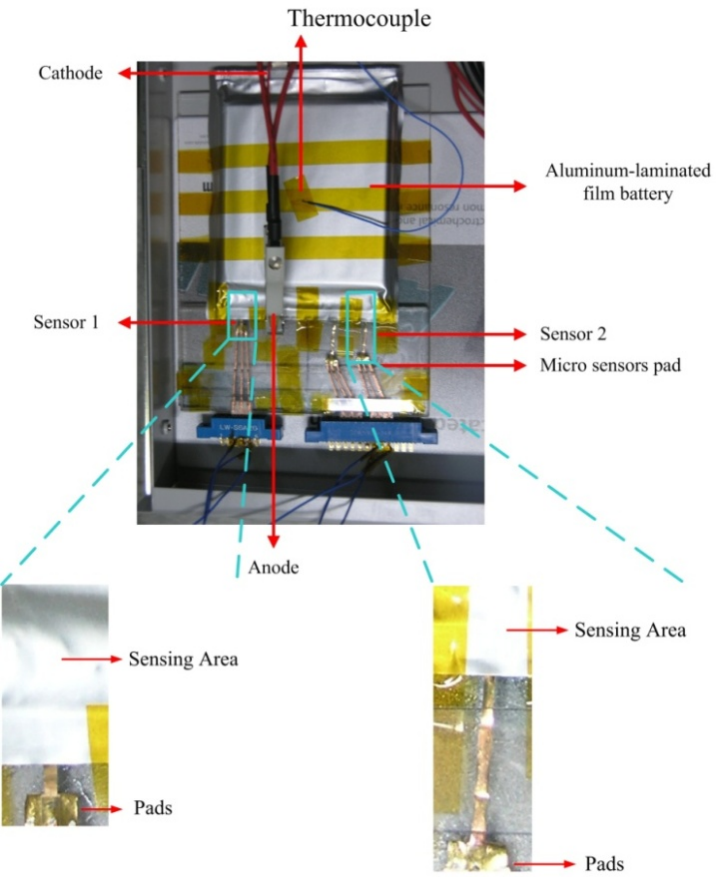
Micro temperature sensor is inserted into lithium-ion secondary battery.

**Figure 11.**
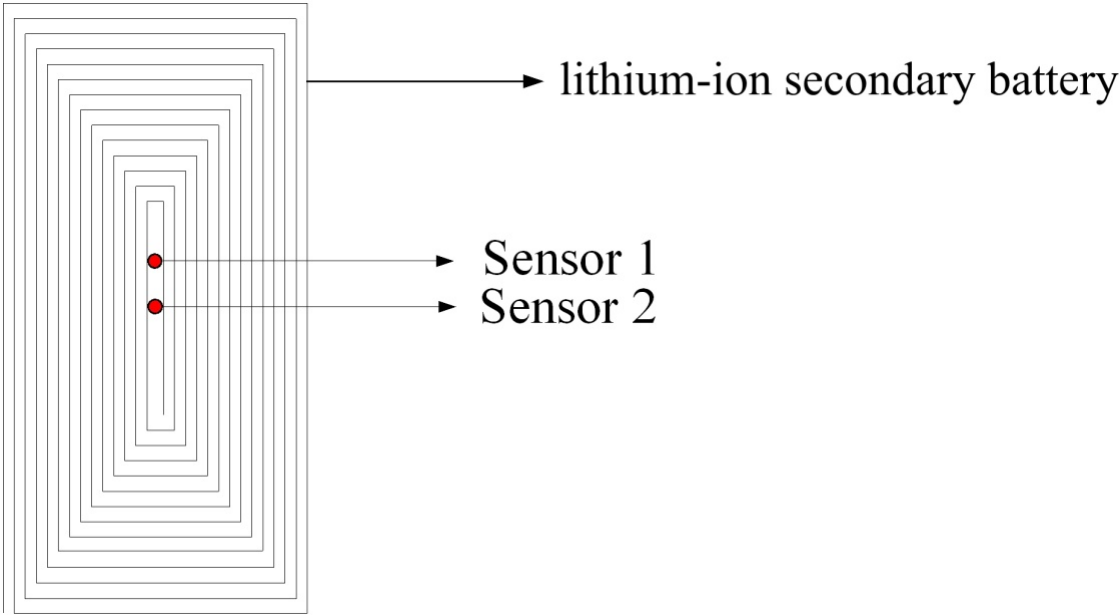
Position of micro temperature sensors in lithium-ion secondary battery.

The micro temperature sensors are inserted into a lithium-ion secondary battery, as shown in Figures 10 and 11. A thermocouple attached to the outer surface of the battery measured the surface temperature. The signals from the micro sensors and the thermocouple were picked up by the Data Acquisition system GBT-2211.

**Figure 12.**
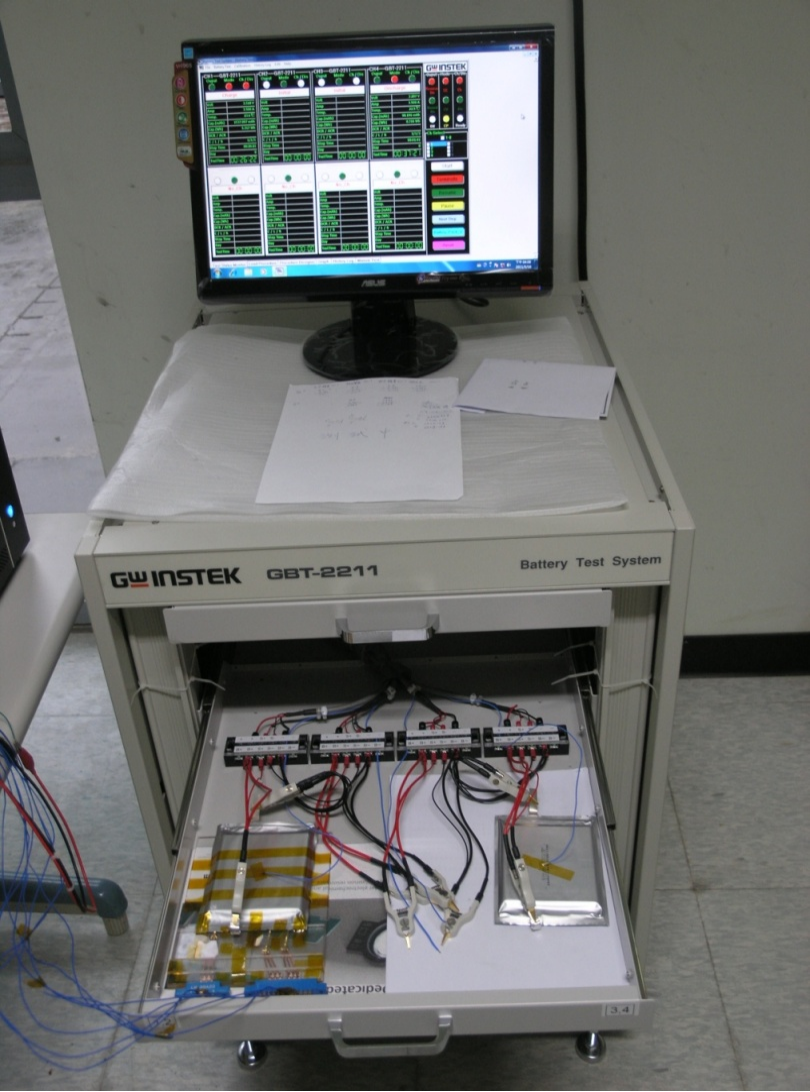
GBT-2211 is used to charge and discharge and lithium-ion secondary battery.

**Figure 13.**
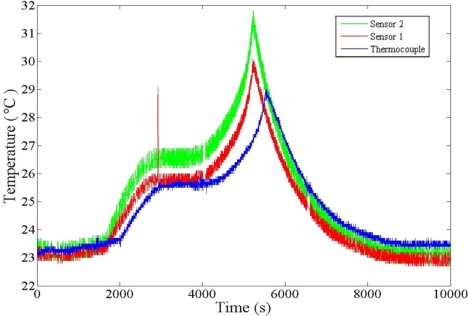
Temperature curve during 1C charging and discharging.

Figure 12 shows the battery tester used in charging and discharging the lithium-ion battery. Figure 13 plots the temperature curve of the thermocouple and the micro temperature sensors. The three curves are all mutually consistent. The temperatures vary among different positions in the lithium-ion secondary battery. The inner temperature changes more rapidly than the outer temperature. At the peak, the inner temperature is 2 °C higher than the outer one.

## 4. Conclusions

In this study, parylene is selected as a flexible material to fabricate micro temperature sensors. The strength of the micro temperature sensor suffices for a lithium-ion secondary battery. The *in situ* measurements of temperature are picked up successfully. The results demonstrate that when the lithium battery 1C charging and discharging reactions occur in the battery, the inner temperature is 2 °C higher than the outer one.
